# Comparing Left Ventricular Structure and Functions in End-Stage Renal Disease Using Conventional Echocardiography and 2D Speckle Tracking Echocardiography

**DOI:** 10.7759/cureus.62926

**Published:** 2024-06-22

**Authors:** Shimaa A Habib, Asmaa A Hasan, Ola Hassan Abd Elaziz, Amal H Ibrahim, Tamer A Ali, Fatma M Kotb, Hani Khalaf, Mohamad A Omar, Ibrahim F Said, Attia M Shokr, Abdelaal A Elkhouly, Ahmed A. Faheem, Ahmed Mohamed Abbas, Tarek A Dabash, Mohamed M Hefzy, Ahmed A Aboomar

**Affiliations:** 1 Department of Cardiology, Faculty of Medicine, Al-Azhar University, Cairo, EGY; 2 Department of Internal Medicine, Nephrology Unit, Faculty of Medicine, Al-Azhar University, Cairo, EGY; 3 Department of Urology, Faculty of Medicine, Al-Azhar University, Cairo, EGY; 4 Department of Internal Medicine, Faculty of Medicine, Al-Azhar University, Cairo, EGY; 5 Department of Cardiology, Faculty of Medicine, Al-Azhar University, Damietta, EGY; 6 Department of Cardiology, Islamic Cardiac Center, Al-Azhar University, Cairo, EGY; 7 Department of Urology and Nephrology, National Institute of Nephrology, Cairo, EGY; 8 Department of Nephrology, Medcare Hospital, Sharjah, ARE; 9 Department of Internal Medicine, Nephrology Unit, Faculty Medicine, Tanta University, Tanta, EGY

**Keywords:** hemodialysis (hd), dialysis, speckle tracking echocardiography, echocardiographic left ventricular hypertrophy, end-stage renal disease

## Abstract

Background: Patients on hemodialysis (HD) are prone to various cardiovascular complications. Two-dimensional speckle tracking echocardiography (2D STE) is an innovative technique for early myocardial dysfunction detection, even with normal ejection fraction (EF).

Objective: We aim to detect left ventricle (LV) dysfunction in regular hemodialysis patients using 2D STE compared to traditional echocardiography.

Methods: The study comprised 30 patients with end-stage renal disease (ESRD), subdivided according to left ventricular mass index (LVMI) into group 1 with left ventricular hypertrophy (LVH) (n=19) and group 2 without LVH (n=11). Another 30 healthy control subjects were recruited as group 3. The EF, average systolic velocity (Sa), and 2D LV strain were taken as measures of LV systolic function. The indicators for diastolic function included the E/A ratio and E velocity/peak early diastolic velocity.

Results: Regarding the parameters of LV systolic and diastolic functions assessed by traditional echocardiography, we found no significant difference between groups 1 and 2. However, using 2D STE, we observed significant differences in the average Sa velocity (p=0.025), average LV strain (p=0.03), 2D global longitudinal strain (GLS) (p=0.03), E/Ea (p=0.003), and LV myocardial performance index (MPI) (p=0.006). Also, a significant positive correlation was found between LVMI and left ventricular end-diastolic diameter (LVEDD) (p<0.01, r=0.63), EF measured by 2D (p=0.034, r=0.39), mitral E/A ratio (p=0.03, r=0.49), and mitral E/Ea (p<0.01, r=0.72). There was a significantly strong negative correlation between LVMI and 2D average LV strain (p=0.034, r=-0.39).

Conclusion: We concluded that 2D STE is more sensitive than a conventional echo in detecting early LV systolic and diastolic dysfunction even in patients with normal EF.

## Introduction

Cardiovascular problems are considered the main cause of death in end-stage renal disease (ESRD) patients who are on regular hemodialysis (HD) [1]. These patients are prone to cardiovascular complications due to hemodynamic disturbances such as hypertension, fluid and sodium retention, and anemia, as well as inflammation and endocrine disorders [2]. The initial cardiovascular abnormality starts with left ventricular hypertrophy (LVH), which occurs to compensate for volume and pressure overload [3]. However, with time, pathological LVH could result from collagen deposition, fibrosis, and calcification [4]. The classic classification of LVH into concentric or eccentric is a challenge in ESRD patients owing to the cyclic variations in extracellular fluid volume and electrolyte balance [5]. Previous research has highlighted LVH and systolic and diastolic dysfunction as independent predictors of death in ESRD patients [6]. However, in the early stages of uremia, detection of LV systolic dysfunction is challenging as the parameters of traditional echocardiography such as ejection fraction (EF) and fraction shortening (FS) are often normal. Moreover, these parameters do not provide an insight into the regional systolic function [4,7,8]. This is unlike tissue Doppler imaging (TDI), which can assess the regional systolic function of LV by measuring strain, strain rate, tissue velocities, and displacement. One of the emerging techniques that caught attention in recent years was the two-dimensional speckle tracking echocardiography (2D STE), which can evaluate the regional systolic function and can detect early changes in myocardial function [9,10]. Therefore, we hypothesized that 2D STE can detect the subtle impairment of LV systolic function in uremic patients with normal LVEF.

## Materials and methods

Our study was an observational study that included 30 patients with ESRD and 30 healthy controls. Cases were divided into two groups: group 1 (19 patients) who had ESRD plus LVH and group 2 (11 patients) who had ESRD without LVH. We recruited the cases from the Nephrology Department between January 2021 and September 2022. Our study followed the Helsinki Declaration principles, and ethical approval was obtained from the institutional review board. Written informed consent was obtained from every patient before the inclusion. Protocols and written informed consent for all participants were approved by the Research Ethics Committee of Al-Azhar University Institutional Review Board (IRB) (RHDIRB/2018122001). We recruited the patients according to the following criteria: patients with ESRD who had a glomerular filtration rate below 15 mL/minute/1.73 m^2^ and were on hemodialysis three times/week [11]. The exclusion criteria include any patients with diabetes mellitus, hypertension, and/or heart disease.

Data collection and sample size determination

**Figure 1:**
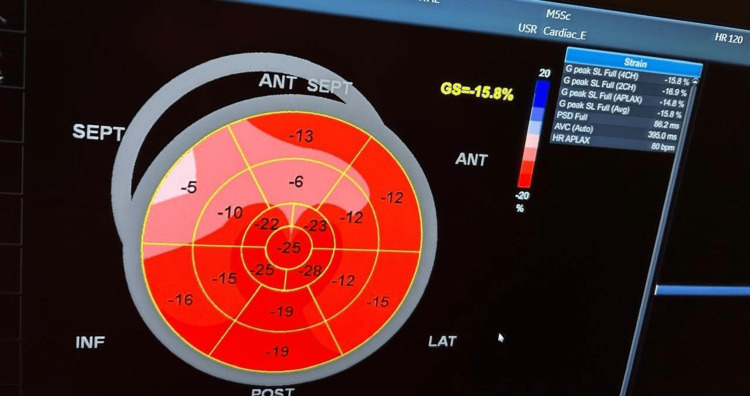
Bull's-eye 2D speckle tracking in a patient with ESRD with LVH ESRD: end-stage renal disease, LVH: left ventricular hypertrophy

**Figure 2:**
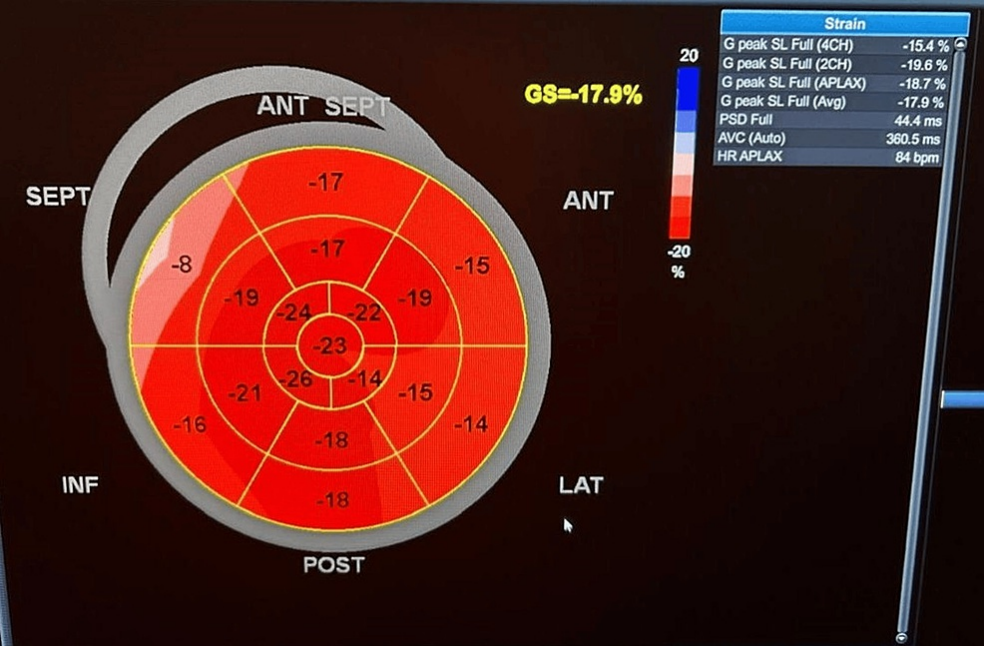
Bull's-eye 2D speckle tracking in a patient with ESRD without LVH ESRD: end-stage renal disease, LVH: left ventricular hypertrophy

All patients and control subjects underwent the transthoracic echocardiography (TTE) examinations. Utilizing the GE system XDclear 9, matrix probe M3S multifrequency 2.5 MHz, with the capacity of TDI and greyscale recording for speckle tracking analysis, the measurements were calculated during three cardiac cycles. The size and functions of the left ventricle were measured using typical Doppler measurements of mitral valve early diastolic velocity (MV E vel), late diastolic velocity (MV A vel), E/A ratio, and deceleration time (DT) (Figure 1 and Figure 2).

The bull's-eye plot, much like the strain graph, is a visualized version of data collected from strain imaging echocardiography. It gives you information on how well segments of the left ventricle wall contract. The contractility is depicted both as colors and global longitudinal strain (GLS) values.

We used the modified Penn formula to calculate LV mass [3]. Cornell criteria for LVH identified a left ventricular mass index (LVMI) of >50 g/m^2^ in males and >47 g/m^2^ in females. As an indicator of the LV geometric pattern, we determined the relative wall thickness (RWT) ((interventricular septum thickness (IVS) or posterior wall thickness (PWT))/LV end-diastolic diameter) (concentric LVH, RWT0.45; eccentric LVH, RWT0.45). Apical four- and two-chamber views provided us with TDI. For collecting data, we gathered three full cardiac cycles and recorded them in a cine loop format. For an acquisition frame rate of more than 90 frames/second, we set the image sector width as narrowly as we could. Mitral annular systolic velocity (Sa) and early diastolic velocity (Ea) were obtained at inferoseptal, lateral, inferior, and anterior annular sites using pulsed wave tissue Doppler. The average values and the E/Ea average ratio were then calculated. The longitudinal strain of the LV was also assessed by 2D STE analysis utilizing QRS onset as the reference point, using a commercially available strain software package using echoPac version 210 (GE Healthcare, Horten, Norway).

To obtain a statistically significant sample size, we used a simple sampling method. The final sample size required for this study is 58 with a confidence level of 95% (Z-score=1.96) (obtained by determining the sample size, the confidence interval, the confidence level, and the standard deviation and converting the confidence level into a Z-score).

Statistical analysis

We used SPSS version 16.0 (IBM SPSS Statistics, Armonk, NY) for data analysis. Continuous parametric data were presented as mean and standard deviation. The Kolmogorov-Smirnov test was used to determine whether the data were parametric or not. Using the t-test, the differences between the groups were examined. Categorical data were presented as numbers and percentages and were compared using the chi-square test. Using Pearson and Spearman coefficients of correlation, potential relationships were evaluated. Significant was defined as a p-value ≤ 0.05.

## Results

Our study included 60 individuals divided into three groups: group 1 included 19 patients with ESRD on maintenance hemodialysis with LVH (concentric in 16 (84.2%) patients and eccentric in three (15.8%) patients), group 2 included 11 patients with ESRD and no LVH, and group 3 included 30 age- and sex-matched healthy controls. Clinical and biochemical data are shown in Table 1.

**Table 1:** Baseline clinical and biochemical characteristics of the study population BMI: body mass index, SBP: systolic blood pressure, DBP: diastolic blood pressure, ESRD: end-stage renal disease, LVH: left ventricular hypertrophy

	ESRD + LVH (n=19)	ESRD (n=11)	Controls (n=30)	p-value
Male (number (%))	12 (65%)	7 (64%)	18 (60%)	0.96
Age (years)	58.53±4.6	58.82±5.15	60.12±5.2	0.52
BMI (kg/m^2^)	28.3±5	28.4±5.65	29.25±5.49	0.80
Systolic blood pressure	129±28	129±10	126±18	0.85
Diastolic blood pressure	89±11	85±9	85±8.5	0.32
Hemodialysis duration (months)	45±30	40±30.32	-	0.66
Smoking (number (%))	5 (26.3%)	3 (27.3%)	8 (26.7%)	0.99
Etiology of ESRD				0.56
Chronic glomerulonephritis	6	4	-	
Interstitial nephritis	3	1	-	
Polycystic kidney	3	1	-	
Analgesic nephropathy	1	2	-	
Obstructive nephropathy	1	-	-	
Unknown	5	3	-	
Hemoglobin (g/dL)	8.2±1.2	8.65±1.35	13.4±1.9	<0.001
Urea (mg/dL)	129.3±44.3	127.5±48.4	25.6±10.4	<0.001
Creatinine (mg/dL)	8.3±2	8.3±2.6	1±0.4	<0.001
Cholesterol (mg/dL)	220±55	211.6±52.5	100.34±20.5	<0.001
Triglycerides (mg/dL)	166.9±19.8	163.7±20.4	90±21.3	<0.001
Hemoglobin (g/dL)	8.2±1.2	8.65±1.35	13.4±1.9	<0.001

Comparison between ESRD patients and healthy controls

Patients with ESRD and LVH differed significantly from controls in terms of diastolic interventricular septum thickness (<0.001), left ventricle mass index (<0.001), diastolic left ventricular posterior wall thickness (<0.001), diastolic left ventricular internal dimension (0.0039), and left atrium diameter (<0.001) (Table 2 and Table 3). The relative wall thickness of the left ventricle and the left ventricular internal size during systole showed no significant differences between the two groups. The indicators of left ventricular (LV) systolic and diastolic function were not significantly different between groups 1 and 2 using standard echocardiographic methods.

**Table 2:** Baseline LV echocardiographic parameters using traditional echocardiographic assessment in all studied groups P1: comparison between group 1 and group 2, P2: comparison between group 1 and group 3, P3: comparison between group 2 and group 3 ESRD: end-stage renal disease, LVH: left ventricular hypertrophy, LV: left ventricle, IVSD: interventricular septum thickness in diastole, LVIDd: left ventricular internal dimension in diastole, LVIDs: left ventricular internal dimension in systole, LVPWd: left ventricular posterior wall thickness in diastole, EF: ejection fraction, FS: fraction shortening, LVMI: left ventricle mass index, LVRWT: left ventricle relative wall thickness, LA: left atrium

	ESRD + LVH (n=19)	ESRD (n=11)	Controls (n=30)	p-value	P1	P2	P3
IVSd (cm)	1.19±0.31	0.93±0.13	0.79±0.21	<0.001	0.014	<0.001	0.22
LVIDd (cm)	5.21±0.47	5.1±0.56	4.8±0.36	0.006	0.78	0.006	0.13
LVIDs (cm)	3.45±0.54	3.36±0.6	2.99±0.35	0.003	0.8671	0.0039	0.0714
LVPWd (cm)	1.15±0.31	0.93±0.1	0.84±0.21	0.0001	0.0414	<0.001	0.5221
EF-M mode (%)	60.46±9.73	60.60±8.3	68.71±5.65	0.0005	0.99	0.0015	0.0105
EF-2D	51.45±9.29	52.22±7.23	60±4.36	0.0001	0.95	0.0002	0.0054
% FS	32.64±5.64	33.1±4.52	38.14±5.24	0.0011	0.97	0.002	0.02
LVMI	53.36±9.65	40±9.1	35.36±10.1	<0.001	0.002	<0.001	0.38
LVRWT	0.44±0.12	0.37±0.06	0.35±0.07	0.0035	0.09	0.002	0.79
LA diameter (cm)	4.16±0.68	4.05±0.86	3.37±0.32	<0.001	0.87	<0.001	0.003

**Table 3:** LV echocardiographic parameters using 2D speckle tracking echocardiography in all studied groups P1: comparison between group 1 and group 2, P2: comparison between group 1 and group 3, P3: comparison between group 2 and group 3 ESRD: end-stage renal disease, LVH: left ventricular hypertrophy, LV: left ventricle, MV: mitral valve, E vel: early diastolic velocity, A vel: late diastolic or atrial velocity, TDI: tissue Doppler imaging, Sa: myocardial systolic excursion velocity, Ea: myocardial early diastolic excursion velocity, Aa: myocardial late diastolic or atrial excursion velocity, GLS: global longitudinal strain

	ESRD + LVH (n=19)	ESRD (n=11)	Controls (n=30)	p-value	P1	P2	P3
MV E vel (m/s)	0.91±0.25	0.72±0.18	0.61±0.09	<0.001	0.013	<0.001	0.17
MV A vel (m/s)	0.67±0.10	0.79±0.19	0.75±0.13	0.043	0.056	0.11	0.68
MV E/A ratio	1.26±0.33	1.02±0.22	0.94± 0.11	<0.001	0.016	<0.001	0.56
Tei index conv.	0.54±0.16	0.65±0.16	0.18±0.13	<0.001	0.12	<0.001	<0.001
Average TDI Sa	5.12±1.65	6.65±1.82	7.54±1.34	<0.001	0.025	0.0005	0.23
Average Ea	8.1±2.7	7.4±2.1	5.4±2	0.0004	0.69	0.0004	0.039
Average Aa	11.7±6.1	12.7±4.6	10±4.60	0.266	0.86	0.49	0.30
Average Ea/Aa	0.77±0.27	0.64±0.24	0.54±0.18	0.0036	0.28	0.002	0.41
LV E/Ea average	14.05±4.13	10.56±2.51	6.25±1.09	<0.001	0.003	<0.001	0.0001
Tei index TDI	0.66±0.16	0.48±0.16	0.4±0.04	<0.001	0.006	<0.001	<0.001
Average LV strain	16.28±2.41	18.11±1.50	20.45±3.78	0.0001	0.03	0.0001	0.08
Average LV 2D GLS %	13.2±4.61	16.69±3.12	18.86±3.18	0.001	0.03	0.0006	0.22

Comparisons between groups 1 and 2 using TDI and 2D STE

Although there were no significant differences in conventional echocardiography, there was a significant difference in the average Sa velocity (p=0.025), average LV strain (p=0.03), 2D GLS (p=0.03), E/Ea (p=0.003), and LV MPI (p=0.006). We found that all three eccentric LVH patients had systolic and diastolic dysfunction (EF: 49±1.5, average Sa: 4.4±1.23, average LV strain: 14.2±2.45, E/A: 1.7±0.1, E/Ea: 14.3±2.1). The correlations between LVMI and LA diameter (p<0.01, r=0.76), LVEDD (p<0.01, r=0.63), EF measured by 2D (p=0.34, r=0.39), fractional shortening (p=0.035, r=0.38), mitral E/A ratio (p=0.03, r=0.49), and mitral E/Ea (p<0.01, r=0.72) were positive and statistically significant, while the correlation between LVMI and 2D average LV strain was strongly negative (p=0.034, r=-0.39) (Table 4).

**Table 4:** Correlations between LV mass and indices of LV systolic and diastolic functions LVMI: left ventricular mass index, LA: left atrium, LVEDD: left ventricular end-diastolic diameter, EF: ejection fraction, LV: left ventricular

	LVMI	
	r	p-value
LA diameter	0.76	<0.001
LVEDD	0.63	<0.001
EF	0.39	0.034
Fractional shortening	0.38	0.035
Mitral E/A ratio	0.03	0.49
Mitral E/Ea	0.72	<0.001
Average LV strain	-0.39	0.034

## Discussion

Some investigations suggested that echocardiographic measures could be used to predict the onset of LV dysfunction in ESRD patients, but they were unable to establish the gradual changes in systolic and diastolic functions over time [4,7,8]. In the current study, using a recent echocardiographic modality such as 2D STE showed that 80% of the patients had LV diastolic dysfunction and 33% of them had LV systolic dysfunction. Among the 30 patients recruited into the current study, about 62% had LVH and increased LVMI. This finding is in line with those reported by Di Lullo et al. [12] who reported that 62% of their patients had LVH after dialysis for 18 months.

However, our findings were relatively lower than those reported by Zoccali et al. [2] who reported a higher percentage of LVH (77%). This difference may be justified in light of different study inclusion criteria; we excluded hypertensive and diabetic patients, which may account for the lower number of patients with LVH, compared to the later study. In group 1, we found that there was a significant increase in the thicknesses of the posterior LV and interventricular walls. The existence of arteriovenous fistulas, secondary anemia, and intravascular volume expansion (salt and fluid loading) are all factors that contribute to volume overload. Hyperphosphatemia, vitamin D insufficiency, and secondary hyperparathyroidism are possible additional variables that control the proliferation and differentiation of cardiac myocytes [13].

Although EF is the index of systolic function in clinical practice, its measurements can be misleading in patients with LVH. This is because it may be maintained (at least in the early stages) by sub-normally functioning parallel sarcomeres. In the current study, we showed that TDI and 2D STE revealed LV systolic dysfunction in ESRD patients with LVH and normal EF. Because these results were very evident in the eccentric LVH, we may conclude that the LV geometry plays a role in systolic dysfunction. Eccentric hypertrophy is linked to systolic dysfunction more than concentric LVH. LV remodeling was identified by Suh et al. [13] as a significant factor in decompensation. They also showed how eccentric remodeling alters LV volume and shape, which disadvantages the heart mechanically and reduces LV pump efficiency. Paoletti et al. [14] showed that eccentric hypertrophy in renal patients was associated with a significant incidence of cardiovascular adverse events. Uncertainty surrounds the long-term consequences of hemodialysis on LV diastolic function.

Hemodialysis dramatically decreased myocardial blood flow, as demonstrated by Bautz et al. [15]. Without epicardial vascular disease, persistent ischemia events can cause LV systolic dysfunction. LV diastolic dysfunction is a result of the coexistence of hypervolemia, hyperdynamic circulation, LVH, and fibrosis. The drop in ventricular preload lowers peak E velocity, which lowers the E/A ratio as a result. The measured decrease in the E/A ratio after HD may be influenced by the reduced preload. Conventional pulsed Doppler technique can assess trans-mitral flow velocities and isovolumic relaxation time (IVRT). However, its use is unfeasible in patients with elevated filling pressures and pseudo-mitral flow. Pulsed Doppler tissue imaging can give more accurate information by assessing cardiac muscle function and velocity. Our results agreed with those reported by Hayashi et al. [16]; using Doppler to assess velocity can give valuable information on LV function in CKD patients. Our results were also in agreement with those reported by Banerjee et al. [17] who reported a high prevalence and poor prognosis of LV dysfunction in these patients.

The widely used 2D echocardiography may underestimate the LV function, which points to a defect in the literature's interpretation. We can precisely quantify the prevalence of LV dysfunction, thanks to current measurement tools. According to Demetgul et al. [18], 2D STE offers early detection of decreased LV function in CKD patients with normal LVEF. Furthermore, de Bie et al. [19] observed a significant frequency of diastolic dysfunction in dialysis patients and stated that the main factors that determined poor diastolic function in these individuals were LV mass and pulse wave velocity.

Our findings corroborated those of Ma et al. [20], who showed that STE could identify the impairment in diastolic function even with normal EF in the CKD group.

The few patients in our study represent a limitation. Larger prospective trials should validate these findings. For a better knowledge of its consequences, we also advise more research into LV function both before and after hemodialysis. It is also advised to perform subgroup analyses on patients who have eccentric LVH or are receiving peritoneal dialysis.

## Conclusions

The present study concluded that LVH and LVMI are prevalent with ESRD on hemodialysis. The presence of LVH may worsen the LV systolic and diastolic functions. Further, we concluded that recent echo modalities such as 2D STE are more sensitive than a conventional echo in the detection of early LV systolic and diastolic dysfunction even in patients with normal EF.

## References

[1] ESfandiar N, Alaei M, Alaei F, Vahidshahi K, Javdani Yecta S (2022). Echocardiographic indices in pediatric chronic kidney disease. Iran J Kidney Dis.

[2] Zoccali C, Benedetto FA, Tripepi G (2006). Left ventricular systolic function monitoring in asymptomatic dialysis patients: a prospective cohort study. J Am Soc Nephrol.

[3] Hayashi SY, Rohani M, Lindholm B (2006). Left ventricular function in patients with chronic kidney disease evaluated by colour tissue Doppler velocity imaging. Nephrol Dial Transplant.

[4] Silberberg JS, Barre PE, Prichard SS, Sniderman AD (1989). Impact of left ventricular hypertrophy on survival in end-stage renal disease. Kidney Int.

[5] Jankowski J, Floege J, Fliser D, Böhm M, Marx N (2021). Cardiovascular disease in chronic kidney disease: pathophysiological insights and therapeutic options. Circulation.

[6] Chen R, Wu X, Shen LJ, Wang B, Ma MM, Yang Y, Zhao BW (2014). Left ventricular myocardial function in hemodialysis and nondialysis uremia patients: a three-dimensional speckle-tracking echocardiography study. PLoS One.

[7] Foley RN, Parfrey PS, Harnett JD, Kent GM, Martin CJ, Murray DC, Barre PE (1995). Clinical and echocardiographic disease in patients starting end-stage renal disease therapy. Kidney Int.

[8] Wang AY, Wang M, Lam CW, Chan IH, Zhang Y, Sanderson JE (2008). Left ventricular filling pressure by Doppler echocardiography in patients with end-stage renal disease. Hypertension.

[9] Taha M, Labib D, Baghdady Y, El-Ghobashy N, Elamragy AA (2022). Subclinical left ventricular dysfunction during systemic lupus erythematosus activity with follow-up after remission - a speckle tracking echocardiographic study. Egypt Rheumatol.

[10] Naseem M, Samir S, Ibrahim IK, Khedr L, Shahba AA (2019). 2-D speckle-tracking assessment of left and right ventricular function in rheumatoid arthritis patients with and without disease activity. J Saudi Heart Assoc.

[11] Ketteler M, Block GA, Evenepoel P (2018). Diagnosis, evaluation, prevention, and treatment of chronic kidney disease-mineral and bone disorder: synopsis of the Kidney Disease: Improving Global Outcomes 2017 clinical practice guideline update. Ann Intern Med.

[12] Di Lullo L, Gorini A, Russo D, Santoboni A, Ronco C (2015). Left ventricular hypertrophy in chronic kidney disease patients: from pathophysiology to treatment. Cardiorenal Med.

[13] Suh SH, Oh TR, Choi HS (2022). Association between left ventricular geometry and renal outcomes in patients with chronic kidney disease: findings from Korean cohort study for outcomes in patients with chronic kidney disease study. Front Cardiovasc Med.

[14] Paoletti E, Cassottana P, Bellino D, Specchia C, Messa P, Cannella G (2002). Left ventricular geometry and adverse cardiovascular events in chronic hemodialysis patients on prolonged therapy with ACE inhibitors. Am J Kidney Dis.

[15] Bautz J, Stypmann J, Reiermann S (2022). Prognostic implication of myocardial perfusion and contractile reserve in end-stage renal disease: A direct comparison of myocardial perfusion scintigraphy and dobutamine stress echocardiography. J Nucl Cardiol.

[16] Hayashi SY, Brodin LA, Alvestrand A (2004). Improvement of cardiac function after haemodialysis. Quantitative evaluation by colour tissue velocity imaging. Nephrol Dial Transplant.

[17] Banerjee D, Rosano G, Herzog CA (2021). Management of heart failure patient with CKD. Clin J Am Soc Nephrol.

[18] Demetgul H, Giray D, Delibas A, Hallioglu O (2018). 2D-speckle tracking echocardiography contributes to early identification of impaired left ventricular myocardial function in children with chronic kidney disease. Cardiol Young.

[19] de Bie MK, Ajmone Marsan N, Gaasbeek A (2012). Left ventricular diastolic dysfunction in dialysis patients assessed by novel speckle tracking strain rate analysis: prevalence and determinants. Int J Nephrol.

[20] Ma W, Liu N, Tong M, Zhou H (2015). Evaluation of left ventricular function in uremic patients by speckle tracking imaging. Cell Biochem Biophys.

